# Validated ^1^H and ^13^C Nuclear Magnetic Resonance Methods for the Quantitative Determination of Glycerol in Drug Injections

**DOI:** 10.3390/molecules23051177

**Published:** 2018-05-15

**Authors:** Jiaxi Lu, Pengli Wang, Qiuying Wang, Yanan Wang, Miaomiao Jiang

**Affiliations:** 1 Institute of Traditional Chinese Medicine Research, Tianjin University of Traditional Chinese Medicine, Tianjin 300193, China; 18712852757@163.com (J.L.); wangpengli2017@126.com (P.W.); wangqiuying16@gmail.com (Q.W.); 2 State Key Laboratory of Bioactive Substance and Function of Natural Medicines, Institute of Materia Medica, Chinese Academy of Medical Sciences & Peking Union Medical College, Beijing 100006, China; wangyanan@imm.ac.cn

**Keywords:** quantitative analysis, ^1^H NMR, ^13^C NMR, glycerol

## Abstract

In the current study, we employed high-resolution proton and carbon nuclear magnetic resonance spectroscopy (^1^H and ^13^C NMR) for quantitative analysis of glycerol in drug injections without any complex pre-treatment or derivatization on samples. The established methods were validated with good specificity, linearity, accuracy, precision, stability, and repeatability. Our results revealed that the contents of glycerol were convenient to calculate directly via the integration ratios of peak areas with an internal standard in ^1^H NMR spectra, while the integration of peak heights were proper for ^13^C NMR in combination with an external calibration of glycerol. The developed methods were both successfully applied in drug injections. Quantitative NMR methods showed an extensive prospect for glycerol determination in various liquid samples.

## 1. Introduction

Glycerol is a simple polyol compound with three hydroxyl groups that are responsible for its solubility in water, hygroscopic, and hyperosmolar nature. As a potent osmotic dehydrating agent, glycerol has been used in medicinal and pharmaceutical preparations with additional effects on stroke, head injury, brain edema and glaucoma to reduce elevated tissue pressure [1,2,3,4]. Hematuria was the only side effect reported in a small proportion of patients treated with glycerol, which was not explicitly recorded in all studies [5]. Although side effects of glycerol are infrequent and seem to be negligible [6], a large amount of it can produce an ethanol-like anesthesia effect, and lead to high glucose and triacylglycerol in blood [7]. Appropriate analytical methods are therefore needed in order to control the officially required specifications of glycerol.

Chemical titration after reaction with sodium periodate as oxidizing agent has been widely adopted in the pharmacopoeia of China, Japan, America, and Europe. However, any compound containing a couple of neighbor hydroxyls or phenolic hydroxyl is able to participate in a redox reaction, resulting in unsatisfactory specificity and accuracy of this method. Analytical chromatography hyphenated with different detections has also been developed for glycerol determination in drug injections. Physical restrictions of glycerol—such as lack of chromophores, high boiling point, and non-volatility—make it difficult to detect by using conventional gas chromatography (GC) and high performance liquid chromatography-ultraviolet (HPLC-UV) techniques, unless assisted with the process of pre-column derivatization [8,9]. Since every single analyte requires its individual elaborative and time-consuming sample derivatization, efficient alternative screening approaches would be preferable. HPLC equipped with an evaporating light scattering detection and amino column has been described as a useful alternative method to determine glycerol [10]; however, amino column is not durable and has a rapid decrease of column efficiency. 

Nuclear magnetic resonance (NMR) spectroscopy is an essential analytical tool used to unambiguously identify known and novel compounds. It has an inherent advantage that the intensity of resonance signal is directly proportional to the number of nuclei [11], which provides a possibility to simultaneously qualify and quantify several molecules in natural samples [12] such as foods [13], plants or herbal remedies [14], and biofluids [15]. Additionally, NMR technique is non-destructive and does not require any complex sample pretreatment. To the extent of our knowledge, NMR method has not been explored for glycerol quantification in the literature. The aim of our study was to develop a rapid and accurate alternative technique to analyze glycerol for compliance with legal requirements. Hence, we investigated for the first time the use of proton and carbon nuclear magnetic resonance spectroscopy (^1^H and ^13^C NMR) for quantitative analyses of glycerol with some advantages in relation of these chemical and chromatographic methods. 

## 2. Results and Discussion

### 2.1. Signal Assignments and the Specificity

Proton NMR spectra of homemade and injection samples in D_2_O showed the presence of methine proton at *δ_H_* 3.77 (1H, m) and two methylene groups at *δ_H_* 3.63 (2H, dd, *J* = 11.7, 6.5 Hz) and *δ_H_* 3.54 (2H, dd, *J* = 11.7, 4.4 Hz), corresponding to proton signals of glycerol. Methyl signal at *δ_H_* 0.00 and ethylene signal at *δ_H_* 6.40 were assigned to the chemical shift reference TSP-*d*_4_ and the internal standard maleic acid, respectively (Table 1). 

**Figure 1:**
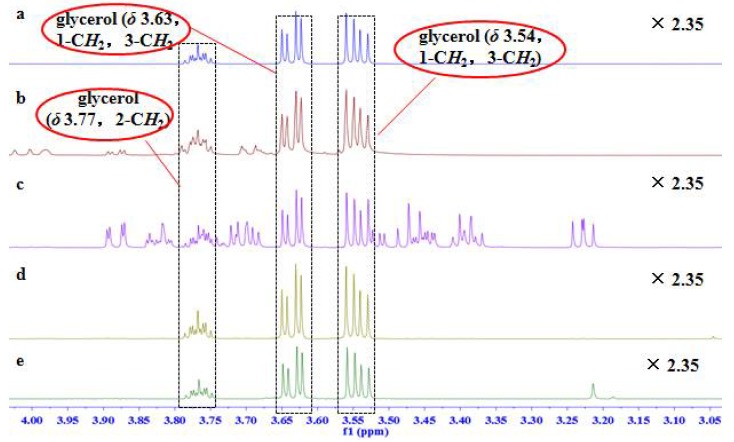
Enlarged ^1^H NMR spectra from *δ* 3.05 to *δ* 4.00 of (**a**) homemade testing sample; (**b**) glycerol fructose and sodium chloride injection (GFS); (**c**) Shenxiong glucose injection (SXG); (**d**) Xiaozhiling injection (XZL); and (**e**) etomidate injectable emulsion (EIE).

**Figure 2:**
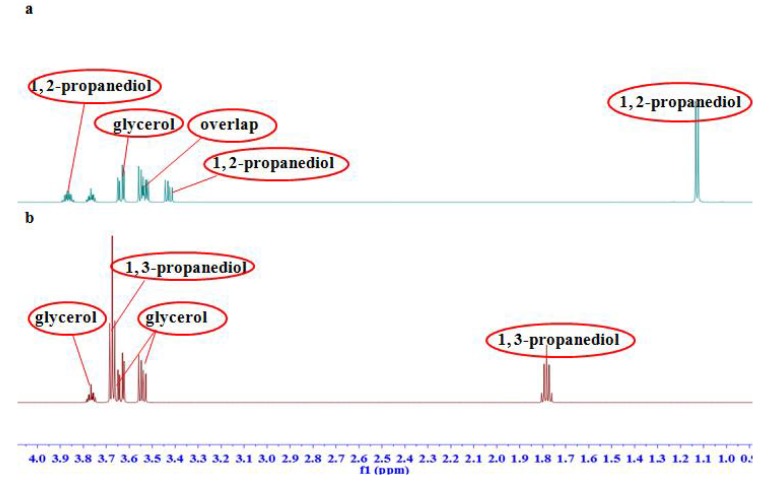
^1^H NMR spectra of (**a**) glycerol and 1,2-propanediol mixture and (**b**) glycerol and 1,3-propanediol mixture.

Enlarged spectra in range of *δ* 3.05–4.00 (Figure 1) indicated the proton signals of glycerol were partially affected by some signals of fructose in the glycerol fructose and sodium chloride injection (GFS) and of glucose in the Shenxiong glucose injection (SXG) injections. Several analogues, such as 1,2-propanediol and 1,3-propanediol, have similar chromatographic behaviors to glycerol, which make them difficult to separate. In ^1^H NMR spectra (Figure 2), the proton signals of 1,3-propanediol and glycerol were totally separated without any overlap, and only one of methylene signals of glycerol was overlapped with that of 1,2-propanediol in the region of *δ_H_* 3.50–3.56.

**Figure 3:**
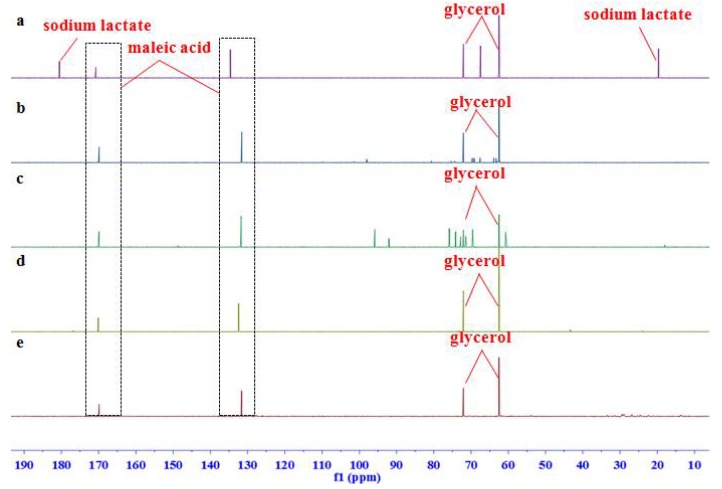
Representative ^13^C NMR spectra of (**a**) homemade testing sample; (**b**) glycerol fructose and sodium chloride injection (GFS); (**c**) Shenxiong glucose injection (SXG); (**d**) Xiaozhiling injection (XZL); and (**e**) etomidate injectable emulsion (EIE).

**Figure 4:**
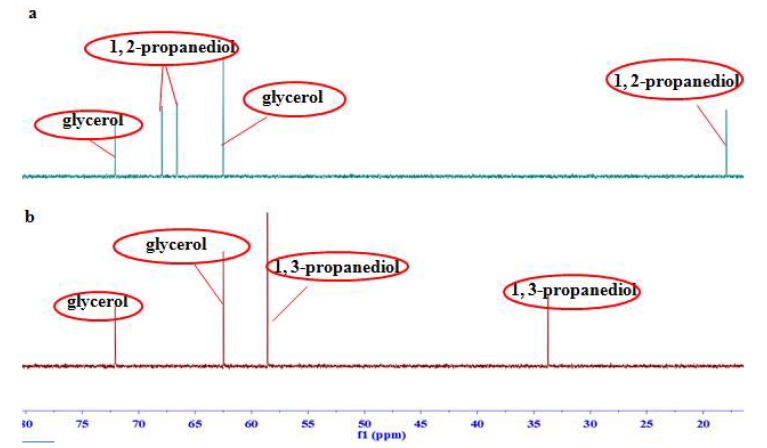
^13^C NMR spectra of (**a**) glycerol and 1,2-propanediol mixture and (**b**) glycerol and 1,3-propanediol mixture.

Since the range of ^13^C spectral width is 20 times larger than that of ^1^H, the problem of overlap in ^1^H NMR spectrum is expected to be reduced in ^13^C NMR spectrum. We thereby recorded the carbon NMR spectra of the samples as well, in which the methine carbon signal at *δ_C_* 73.1 and two methylene signals at *δ_C_* 62.5 were assigned to glycerol and two ethylene signals at *δ_C_* 132.6 and 170.8 were to maleic acid. Moreover, carbon signals of glycerol and the internal standard (maleic acid) were all separate with the signals of other ingredients in the homemade samples, injections, and the analogue mixtures (Figure 3 and Figure 4), which revealed a better specificity of ^13^C NMR spectroscopy than that of ^1^H NMR.

### 2.2. Options for Pulse Sequences

Due to the presence of a very strong solvent signal of H_2_O in drug injections and residual proton signal in deuterated H_2_O, we adopted a solvent-suppression pulse sequence (zgcppr) to acquire ^1^H NMR data with pre-saturation at the H_2_O/HDO solvent frequency before 90° hard pulse. For providing maximum intensity of proton signals, the length of 90° pulse is correspondingly calibrated for each sample, which might vary from sample to sample [16].

Pulse sequence with the effectiveness of solvent suppression can cause some loss of peaks close to the water peak. A particular advantage of ^13^C NMR experiment is the absence of water resonance, and hence, solvent suppression is no longer required. Other factors such as heteronuclear coupling and nuclear Overhauser effect (NOE) enhancement for ^13^C nuclei need to be considered [17]. To ameliorate these problems, we employed an inverse gated-decoupling pulse sequence (zgig) to record ^13^C NMR spectra. In this experiment, ^1^H decoupling is active during ^13^C acquisition, whereas it is switched off during the relaxation delay to suppress the NOE-effect, and thus the acquired spectrum can be integrated.

### 2.3. Options for Acquisition and Post-Processing Conditions

Most of the acquisition parameters are robust and can be varied within wide ranges. For instance, repetition time to acquire a single-scan spectrum depends on the longitudinal relaxation time *T*_1_ of interest signals. Theoretically, five times of the longest *T*_1_ are chosen to measure 99.3% of the equilibrium magnetization. When multiple scans are performed, it will take a long time to record spectrum. On the other hand, it has been shown [18] that for steady-state magnetization, the sensitivity is maximized by setting the repetition rate equal to 1.3*T*_1_. This leads to a signal-to-noise (S/N) ratio that is approximately 1.4 times greater than that obtained using a recycle time of 5*T*_1_ for a given period of data collection. Thus, several reports have also employed shorter relaxation time to yield comparable quantification results. The proton *T*_1_ values of glycerol and maleic acid were determined by inversion-recovery experiment, and the results were shown in Table 2. We subsequently set the relaxation delay on 2.35*T*_1_, at which point magnetization had recovered about 90% and the S/N ratio was also acceptable. Considering the time-consuming acquisition of ^13^C NMR spectra, an empirical value of 10 s was employed as the relaxation delay time in the experiments. To check the relative errors caused by these settings, glycerol samples with two known concentrations were measured and processed with different methods (Table 3).

The interest peaks of glycerol and internal standard were integrated by areas in ^1^H NMR spectra, and glycerol concentration was calculated through direct proportion to the integral area of internal standard or by an external calibration obtained in linearity examination. The relative errors of internal standard calculation method at two concentrations were 0.000 and 0.018, while those of external calibration calculation method were 0.073 and 0.037, indicating that the internal standard calculation method and peak area integration was proper to ^1^H NMR analysis.

Integrations based on peak area and peak height in combination with two calculation methods were employed to determine the content of glycerol in ^13^C NMR spectra. The results showed that the minimum relative errors at two concentrations were 0.004 and 0.014, revealing that peak height integration and external calibration calculation method were alternative for ^13^C NMR analysis.

### 2.4. Method Validation

Using the selected processing conditions, the quantitative NMR methods were validated in sequence of precision, repeatability, stability, accuracy, and linearity (Table 4). The precision was evaluated by six replicate measurements on the testing sample and the relative standard deviation (RSD) values of precision were found to be 0.36% and 0.40% for ^1^H and ^13^C NMR methods, respectively. The repeatability was assessed by analyzing six different solutions independently prepared as the testing sample and the RSD values were 0.55% and 1.48% for ^1^H and ^13^C NMR methods, respectively. The stability was evaluated by analyzing the same sample solution at an interval of every 2 h and the RSD values were 0.35% and 0.96% for ^1^H and ^13^C NMR methods, respectively. Recovery tests were performed to determine the accuracy of quantitative NMR method, and the results showed the average recoveries of glycerol in ^1^H and ^13^C NMR experiments were 95.8% and 101.8% with RSD values of 0.68% and 0.98%, respectively.

**Figure 5:**
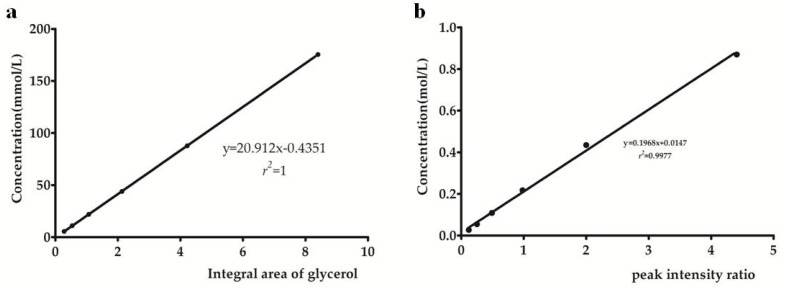
The plots of calibration curves for glycerol quantification (**a**) by ^1^H NMR method and (**b**) by ^13^C NMR method.

Six solutions of glycerol in different concentrations were prepared and analyzed in triplicate to determine the linearity of NMR methods. The linear regression equations (correlation coefficients) were *y* = 20.912*x* − 0.435 (*r*^2^ = 1.0000) and *y* = 0.1968*x* + 0.0147 (*r*^2^ = 0.9977) for the established ^1^H and ^13^C NMR methods, respectively (Figure 5). In addition, LOD and LOQ for glycerol were determined to be 0.015 and 0.045 mM for ^1^H NMR method, as well as 0.16 and 0.48 mM for ^13^C NMR method. These results indicated good precision, repeatability, stability, accuracy, and linearity of developed ^1^H and ^13^C NMR methods, along with lower LOD and LOQ of ^1^H NMR method than those of ^13^C NMR method.

### 2.5. NMR Quantification for Glycerol in Injection Samples

**Figure 6:**
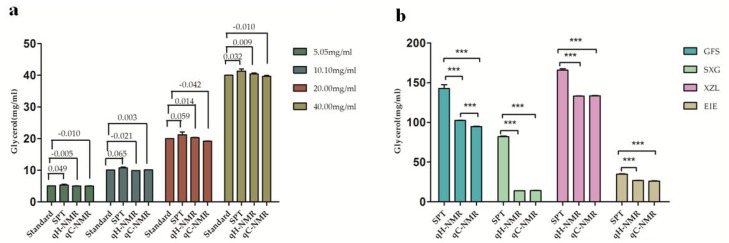
Clycerol contents determined by sodium periodate titration (SPR), ^1^H NMR (qH-NMR) and ^13^C NMR (qC-NMR) methods (**a**) in the homemade testing samples with glycerol at four concentrations (the relative errors were marks on the histograms); (**b**) in the injection samples, including glycerol fructose and sodium chloride injection (GFS), Shenxiong glucose injection (SXG), Xiaozhiling injection (XZL), and etomidate injectable emulsion (EIE). *** *p* < 0.001.

The contents of glycerol in homemade and injection samples were determined by the proposed NMR methods as well as sodium periodate titration (SPR) method. Each sample was analyzed in triplicate and the results were summarized in Figure 6. According to the known amounts of glycerol in homemade samples, the relative errors of three methods were calculated and the results suggested that chemical titration method was less accurate than NMR methods (Figure 6a). 

The average glycerol contents in SXG, GFS, XZL, and EIE injections were estimated to be 13.82 ± 0.04 mg/mL, 102.41 ± 0.40 mg/mL, 133.09 ± 0.27 mg/mL, and 26.86 ± 0.21 mg/mL by employing ^1^H NMR spectra, and to be 14.10 ± 0.20 mg/mL, 94.86 ± 0.50 mg/mL, 133.54 ± 0.38 mg/mL, and 26.03 ± 0.51 mg/mL by ^13^C NMR spectra (Figure 6b). It was found that glycerol contents determined by the titration method were significantly higher (*p* < 0.05) than those measured by NMR techniques, which were far beyond the relative errors of SPR method. One possible explanation for the over-estimation of chemical titration results may be a result of having several reactable compounds in these injections. Periodate oxidation is a selective oxidation reaction that can act with the presence of *O*-dihydroxy or *O*-trihydroxy moiety in the molecular structures. The large amount of glucose in SXG and fructose in GFS probably caused a strong discrepancy between SRT and NMR values, while a relatively small amount of tannic acid in XZL and propylene glycol in EIE showed less influence on the discrepancy between these method results. Moreover, the values of glycerol content in GFS by ^1^H NMR method were found to be higher than those of ^13^C NMR method, which were probably associated with the overlapping signals of glycerol and fructose in the range of *δ* 3.45–4.05. 

## 3. Materials and Methods

### 3.1. Reagents and Materials

Glycerol (99.5%), 3-(trimethylsilyl)propionic-2,2,3,3-*d*_4_ acid sodium salt (TSP-*d*_4_, 98 atom% D), maleic acid (99.94%), sodium l-lactate (99.0%), deuterium oxide (D_2_O, 99.9 atom% D), 1,2-propanediol (99.5%), and 1,3-propanediol (98%) were purchased from Sigma-Aldrich (Steinheim, Germany). Shenxiong glucose injection (SXG, batch no. 20150682) was from Guizhou Jingfeng Injection Co., Ltd. (Guizhou, China). Xiaozhiling injection (XZL, batch no. 15050505) was from Jilin Jian Yisheng Pharmaceutical Co., Ltd. (Jilin, China). Glycerol fructose and sodium chloride injection (GFS, batch no. 1504242) was from Nanjing Chia Tai Tianqing Pharmaceutical (Jiangsu, China). Etomidate injectable emulsion (EIE, batch no. 20150803) was from Jiangsu Nhwa Pharmaceutical Co., Ltd. (Jiangsu, China).

NMR I solution consisted of 0.59 mM TSP-*d*_4_ and 22.67 mM maleic acid in D_2_O, and NMR II solution consisted of 543.11 mM maleic acid in D_2_O.

### 3.2. NMR Measurement

NMR spectra were recorded on a 600 MHz Bruker AVIII HD spectrometer equipped with a 5 mm BBO H&F cryogenic probe. Standard one-dimensional composite pulse sequencing (zgcppr) was used to acquire ^1^H NMR spectra with the following instrumental settings: number of scans = 16; temperature = 298 K; relaxation delay = 16 s; pulse width = 11.5 µs; acquisition time = 1.7039 s; receiver gain = 28; spectral width = 9615.4 Hz; offset = 4125 Hz. ^13^C NMR spectra were acquired by the utilization of the inverse gated-decoupling pulse sequence (zgig) and the acquisition parameters were set as follows: number of scans = 32; temperature = 298 K; relaxation delay = 10 s; pulse width = 6.8250 µs; acquisition time = 3.9716 s; spectral width = 36,057.7 Hz; offset = 4125 Hz. All spectra were manually phased and automatically baseline corrected. Spin-lattice relaxation time (*T*_1_) values of protons in glycerol and maleic acid were measured using a classical inversion recovery pulse sequence with 10 relaxation delays (*τ*) ranging from 0.01 to 20 s.

### 3.3. Quantification

Resonance assignments were based on chemical shifts and spectral databases. Two integral ways, based on peak areas and peak heights, were adopted in conjunction with internal standard or external calibration. Appropriate processing procedures were picked by the relative errors of predicted values compared to the actual mass of glycerol in NMR I and II solutions. The linearity was evaluated by six various contents of glycerol in a range of 5.48–175.4 mM and 27.18–869.6 mM for ^1^H and ^13^C detected experiments, respectively. The limit of detection (LOD) and limit of quantification (LOQ) for glycerol were calculated based on the standard deviation of y-intercept of the regression line and the slope of the calibration curve [11]. Moderate amounts of 1,2-propanediol with glycerol and 1,3-propanediol with glycerol were dissolved in NMR I and II solutions, respectively, which were used to evaluate the specificities of ^1^H and ^13^C NMR methods.

Mixtures of glycerol and sodium lactate were homemade as testing samples for method validation with a glycerol concentration of 21.55 mM in ^1^H NMR experiment and 218.1 mM in ^13^C NMR experiment. The precision of two NMR methods was evaluated by continuously analyzing one testing sample for six times on the same day. One sample was analyzed to determine stability in 0, 2, 4, 6, 8, and 12 h on the same day. The repeatability was determined by analyzing six replicates of testing samples. The amounts of 0.55 mg and 5.05 mg glycerol were added into homemade samples for ^1^H and ^13^C NMR recovery experiments, respectively. The recovery was calculated by (glycerol mass found in glycerol-added testing samples − glycerol mass found in original testing samples)/glycerol mass added × 100%.

For glycerol determination by ^1^H NMR, 100 µL of drug injections were diluted by 2 mL of NMR I solution. For ^13^C NMR analysis, 100 µL of Xiaozhiling injection or glycerol fructose and sodium chloride injection was diluted by 300 µL of ultrapure water and 100 µL of NMR II solution. A volume of 400 µL Shenxiong glucose injection or etomidate injectable emulsion was diluted by 100 µL of NMR II solution. Each mixture was homogenized and 550 µL was transferred into 5 mm NMR tubes.

## 4. Conclusions

Based on quantitative NMR analysis, a reliable method for determination of glycerol in injection has been validated by using maleic acid as internal standard and D_2_O as the NMR solvent. The results of accuracy, linearity, precision, stability, and repeatability emphasize that ^1^H and ^13^C NMR can be used for quantitative determinations of glycerol in injections. Along with the continuous improvement and development of NMR technology, the established methods are probably an alternative for various applications of glycerol in solutions, such as pharmaceutical quality control, food additives, and biofuels.

## Figures and Tables

**Table 1:** Resonance signal assignments of glycerol, maleic acid, 1,2-propanediol, and 1,3-propanediol.

Compound	Structure	Number	^1^H	^13^C
glycerol	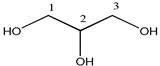	1, 3	3.63 (2H, dd), 3.54 (2H, dd)	62.5
		2	3.77 (1H, m)	73.1
1,2-propanediol	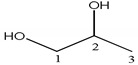	1	3.52 (1H, dd), 3.44 (1H, dd)	67.9
		2	3.86 (1H, m)	66.6
		3	1.13 (3H, d)	17.9
1,3-propanediol	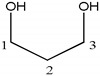	1, 3	3.67 (4H, t)	58.6
		2	1.78 (2H, m)	33.7
maleic acid	HOOCCH=CHCOOH	–CH=CH–	6.40 (s)	132.6
		–COOH		170.8

**Table 2:** Proton *T*_1_ values of glycerol and maleic acid (*n* = 3)

Compound (Chemical Shift)	*T*_1_ (s)	Mean ± SD (s)	RSD (%)
glycerol (*δ* 3.52)	2.29	2.29 ± 0.00	0.22
	2.28		
	2.29		
glycerol (*δ* 3.63)	2.24	2.25 ± 0.00	0.42
	2.25		
	2.26		
glycerol (*δ* 3.75)	4.67	4.86 ± 0.17	3.56
	4.93		
	5.00		
maleic acid (*δ* 6.40)	6.77	6.78 ± 0.02	0.25
	6.78		
	6.78		

**Table 3:** Relative errors of glycerol amounts predicted by ^1^H and ^13^C NMR methods

Detected Atom	Integral Way	Calculated Method	Actual Mass (mg)	Testing Mass (mg)	Absolute Error	Relative Error
^1^H	peak area	internal standard	0.55	0.55	0.00	0.000
			1.09	1.11	0.02	0.018
		external calibration	0.55	0.51	−0.04	0.073
			1.09	1.05	−0.04	0.037
^13^C	peak area	internal standard	5.02	4.78	−0.24	0.048
			10.04	9.81	−0.23	0.023
		external calibration	5.02	4.56	−0.46	0.092
			10.04	8.91	−1.13	0.113
	peak height	internal standard	5.02	4.79	−0.23	0.046
			10.04	10.24	0.20	0.020
		external calibration	5.02	5.00	−0.02	0.004
			10.04	9.90	−0.14	0.014

**Table 4:** Validation results for NMR techniques of glycerol (*n* = 6)

Detected Atom	^1^H	^13^C
integral way	peak area	peak height
calculated method	internal standard	external calibration
linear regression equation	*y* = 20.912*x* − 0.4351	*y* = 0.1968*x* + 0.0147
correlation coefficient (*r*^2^)	1.0000	0.9977
standard deviation of y-intercept	0.094	0.087
LOD (mM)	0.015	0.16
LOQ (mM)	0.045	0.48
precision (RSD %)	0.36	0.40
stability (RSD %)	0.35	0.96
repeatability (RSD %)	0.55	1.48
recovery rate (RSD %)	95.8 (0.68%)	101.8 (0.98%)
